# How Tubercles Destroy

**Published:** 1878-05

**Authors:** 


Tubercles enlarge and are softened by debility of body, long protracted. Whatever then has a debilitating effect on the body, whether of a mental, moral, or physical nature, is the match which fires the magazine of life and burns it to ashes. Whatever keeps the body in a debilitated condition for weeks together, is capable of softening tubercles. If there be a great many tubercles of about the same age, as it were, any debilitating cause, acting for a comparatively short time, commences the decay, which, from the number of tubercles, soon becomes general, and the constitution fails rapidly. This is rapid consumption. It is like a spark applied to a wooden tenement which has been standing for half a century—every inch of wood is a tinder-box.

The evidence of softening tubercle is the spitting up of mouthfuls of yellow matter, which falls lumpily or heavily on the floor, with uneven edges, just as if one had been chewing a rag or piece of paper somewhat soft, and thrown it on the floor. If spit in water it sinks rapidly to the bottom, or if spit into a cup where there is but a spoonful or two of water, and the cup is tilted, the contents run rapidly from side to side. When an ulcer breaks or the lungs are decaying rapidly, the matter expectorated is not unlike thick rich cream.

A truth is about being stated, whose importance is such, that the whole civilized world should keep it in remembrance. As in a tree there may be a single cluster of apples, so in the lungs there may be but a single cluster of tubercles, and the remainder of the lungs may be perfectly sound. Or there may be two clusters or a dozen; each cluster may be a large or a small one; or they may be of various sizes. The symptoms of a ripening cluster are, first, a slight unfrequent cough; then more decided, still dry; next a little mucus comes; soon a large and free expectoration of yellowish matter is observed. If the cluster be large, this yellow matter is not brought away fast enough, and it is reabsorbed into the system. This reabsorption—this mingling of the matter of decayed lungs with the blood again—gives fever, hectic, night-sweats. As soon as the decayed matter of tubercle is removed, the patient begins to get better; the cough has disappeared in great part, if not wholly, the appetite improves, strength returns, flesh is gained, and the man may live half a century.

Whatever was done remedially at the time when the matter was about got rid of, gets the credit of having cured a man in the very last stages of consumption. The ignorant administrator and the more happy recipient, are willing enough to give the remedy the credit, and with all due formality a magistrate is sought, the declaration written, the hat pulled off, the bible procured, the hand held up, the head bowed, the deponent there affirming that

